# Comparative pharmacokinetics of new curcumin preparations and evidence for increased bioavailability in healthy adult participants 

**DOI:** 10.5414/CP204257

**Published:** 2022-10-24

**Authors:** Akiko Hirose, Yoshitaka Kuwabara, Yoko Kanai, Chieko Kato, Yuji Makino, Fukumoto Yoshi, Kazumoto Sasaki

**Affiliations:** 1 Department of R&D, Theravalues Corp.,; 2 CRO Business Div., Apo Plus Station Co. Ltd.,; 3 Institute for Pharmaceutical Research, Musashino University, Tokyo, and; 4 Sasaki Memorial Hospital, Tokorozawa, Saitama, Japan

**Keywords:** curcumin, bioavailability, clinical trial

## Abstract

Objective: Theracurmin, which contains the curcumin composition, CR-033P, has been demonstrated to be highly bioavailable. To compare the pharmacokinetics of the three compositions, CR-033P, CR-043P using modified starch as an alternative to the dispersant gum ghatti used in the CR-033P, and TS-P1 containing the newly developed amorphous curcumin, a randomized double-blind crossover study (3-way, 3-period) was conducted. Materials and methods: A single dose of the curcumin capsules (TS-P1 45 mg, CR-033P 90 mg, and CR-043P 90 mg) was administered to healthy adult participants. Blood sampling was performed 24 hours after capsule administration, and the plasma concentration of total curcumin was determined using high-performance liquid chromatography coupled with tandem mass spectrometry. Results: TS-P1 and CR-043P tended to have a slightly lower area under the concentration time curve (AUC) _0–24h_ than CR-033P, while TS-P1 displayed bioequivalence to CR-043P. Further, TS-P1 displayed bioequivalence to CR-033P in terms of AUC_0–12h_, while that of CR-043P tended to be lower than that of CR-033P. TS-P1 had a higher AUC_0–12h_ than CR-043P. A statistically significant difference (p < 0.001) was found between the preparations in terms of C_max_. TS-P1 tended to have a higher C_max_ than CR-033P, CR-043P tended to have a slightly lower C_max_ than CR-033P, and TS-P1 tended to have a higher C_max_ than CR-043P. Conclusion: The newly developed TS-P1 composition seemed to display similar curcumin systemic exposure except for a higher plasma concentration than the CR-033P composition. Further, only a few significant differences were found between CR-043P and CR-033P.

Trial Registry: UMIN Clinical Trials Registry; UMIN Clinical Trial Registration Number: UMIN000042720 

**What is known about this subject**

- Theracurmin, which contains curcumin compositions of CR-033P or CR-031P, has shown significantly enhanced absorption compared to curcumin powder.

**What this study adds**

- In this study, we compared the human PK profiles of a newly developed amorphous curcumin composition, TS-P1, with CR-033P composition in a randomized crossover study. The PK profiles were compared to those of CR-043P.
- The TS-P1 composition displayed similar systemic exposure and higher curcumin concentration than the CR-033P composition. Further, only a few significant differences were found between the CR-043P and CR-033P compositions.

## Introduction 

Curcumin, a natural polyphenolic compound derived from turmeric (*Curcuma longa* L.), is the major bioactive compound exhibiting various nutritional and pharmacological activities. Studies have reported the beneficial effects of curcumin on neurological, cardiovascular, lung, metabolic, and liver diseases, mainly through anti-inflammatory and antioxidant mechanisms [1, 2, 3]. The role of curcumin as an anticancer agent has also been suggested as it exerts immunomodulatory effects in a wide range of tumors, such as breast, hematological, gastric, colorectal, pancreatic, hepatic, and prostate cancers [4, 5]. 

Despite its promising biological activity, the clinical application of curcumin has been limited owing to its low bioavailability. Curcumin is almost insoluble in water and is susceptible to degradation, especially under alkaline conditions. Furthermore, curcumin undergoes extensive metabolic processes, such as glucuronidation and sulfation, for excretion. Several formulations have been developed to overcome the low bioavailability of phospholipid complexes, microemulsions, liposomes, polymeric micelles, and nanoparticles. Theracurmin, which contains curcumin dispersed as colloidal submicron particles, is one of such bioavailable curcumin compositions [6]. 

Recently, Chung et al. [7] administered two types of theracurmin composition-containing preparations: CR-031P capsule (curcumin 30 mg/capsule) and CR-033P capsule (curcumin 90 mg/capsule) to humans and evaluated their pharmacokinetics (C_max_, AUC_0–12h_). When the same dose (90 mg) was administered, the C_max_ and AUC_0–12h_ of CR-033P and CR-031P were found to be similar. Further, the point-estimated ratios of CR-033P to unformulated curcumin at C_max_ and AUC_0–12h_ were 20.5-fold and 42.6-fold, respectively. In contrast, the ratios of CR-031P were 18.4-fold and 35.9-fold, respectively [7]. 

In this study, we further investigated the human pharmacokinetics of two new curcumin compositions: TS-P1, which contains amorphous curcumin (45 mg), and CR-043P, which contains submicron crystalline curcumin (90 mg) and modified starch (as an alternative for gum ghatti used in CR-033P). The TS-P1 composition was designed to enhance curcumin oral absorption owing to its expected amorphous superiority in solubility. Furthermore, the CR-043P composition is expected to be accepted in more countries; as the dispersant, gum ghatti, is restricted in some countries. The pharmacokinetic parameters of TS-P1 and CR-043P were compared with those of CR-033P. 

Since we expected that TS-P1 capsule would be bioequivalent to half dose of CR-033P or CR-043P capsule, we conducted a study based on “Guideline for bioequivalence studies of generic products (PMDA, Japan: March 19, 2020) using TS-P1 capsule with the half dose of CR capsules. 

## Materials and methods 

### Ethics 

This study was conducted in accordance with the Declaration of Helsinki (revised in October 2013) and the Ethical Guidelines for Medical and Health Research Involving Human Subjects (partially amended on February 28, 2017), with the protection of the rights of the participants considered. 

The study was approved by the Ethics Committee of Kanazawa Bunko Hospital (date of approval: December 1, 2020) and registered and published at the UMIN Clinical Trials Registry (UMIN Clinical Trial Registration Number: UMIN000042720), with sufficient attention dedicated to safety. The clinical part of the study was conducted at Sasaki Memorial Hospital under the responsibility of the chairman, Dr. Kazumoto Sasaki. The principal investigator fully explained the content of the study to the participants, who signed an informed consent form to indicate their participation at free will. 

### Sample preparation 

The three investigational preparations included TS-P1 capsule containing amorphous curcumin (45 mg) and other excipients, such as diluents and lubricants; CR-043P capsule containing submicron crystalline curcumin (90 mg) and modified starch (as an alternative for gum ghatti used in the CR-033P formulation) but with the same ingredients as CR-033P capsules containing submicron crystalline curcumin (90 mg) along with other excipients, such as diluents and lubricants. Placebo capsules were also prepared. The capsules were size #1, milky-white, tasteless, odorless, and indistinguishable in appearance. The manufacturing method of the CR-033P composition or CR-043P composition was the same as previously published [8], whereas that of TS-P1 was proprietary. 

### Participants 

**Figure 1.**
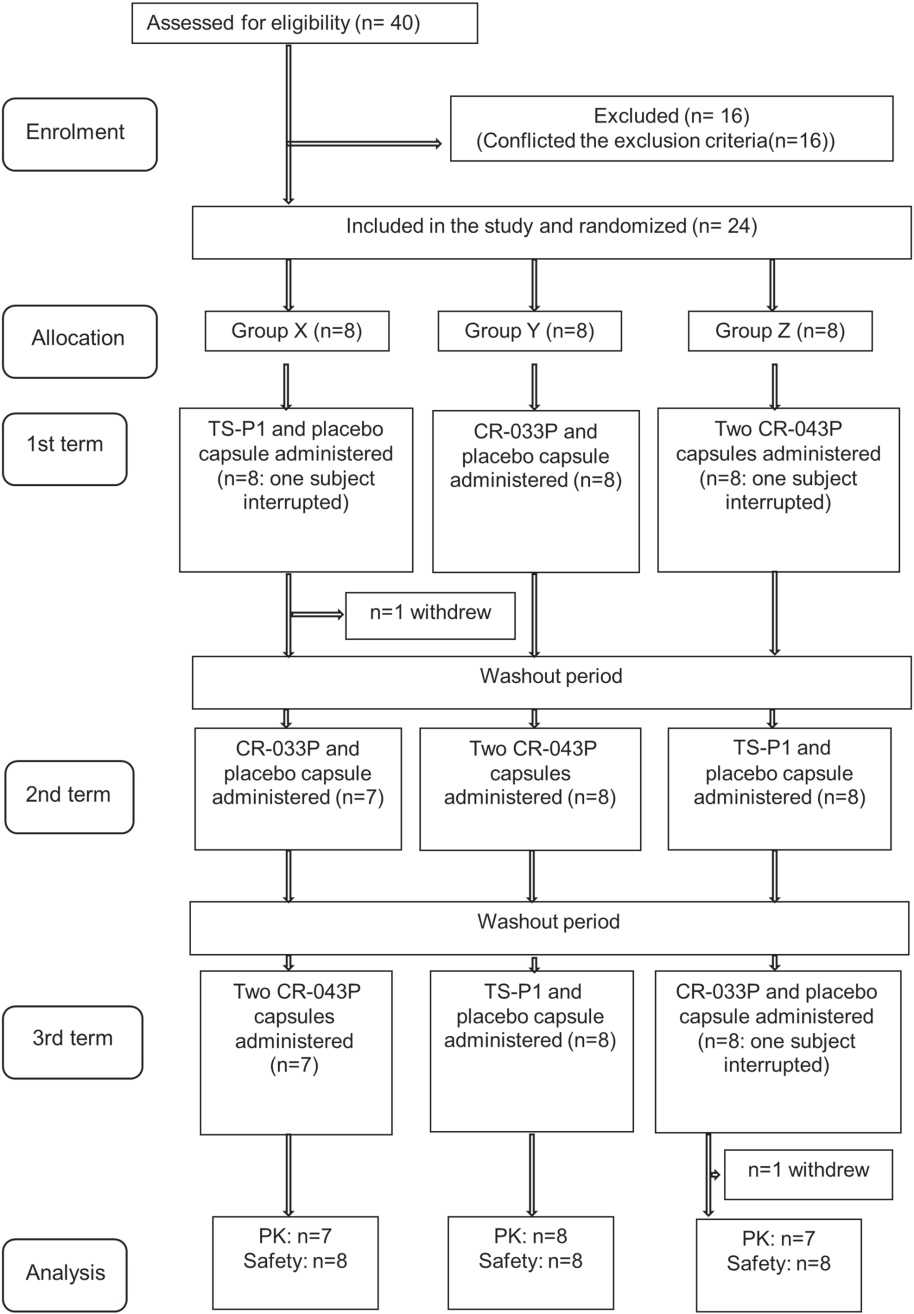
Consort diagram – participant flow chart.

Informed consent was obtained from the 40 participants prior to the study. The participants underwent screening, including medical history, vital signs, clinical laboratory tests, and physical examinations (Table 1). The 24 participants who satisfied all inclusion criteria (Supplemental Table 1) and did not meet any of the exclusion criteria (Supplemental Table 2) were enrolled in the study. All were randomly allocated to 3 groups (X, Y, and Z). Of the 24 participants, 22 completed the treatment, while 2 discontinued treatment during the study (Figure 1). The study participants were healthy Japanese men and women aged 20 – 59 years at the time of agreeing to participate. These participants agreed to visit the study facility on the planned date and stay at the facility for 3 days and 2 nights. 

### Study design 

This was a double-blind, randomized crossover study (3 preparations, 3 periods), which was designed basically in accordance with “Guideline for bioequivalence studies of generic products (PMDA, Japan: March 19, 2020)”. The participants were divided into 3 groups, as shown in Figure 1. Each group was administered 1 of the 3 preparations (CR-033P capsule, CR-043P capsule, and TS-P1 capsule) every 7 days. The TS-P1 group was administered 1 TS-P1 capsule and 1 placebo capsule, the CR-043P group was administered 2 CR-043P capsules, and the CR-033P group was administered 1 CR-033P capsule and 1 placebo capsule. Participants refrained from consuming curcumin-containing food supplements or foods (turmeric, curry, etc.) for 3 days prior to the study. During the study, the participants were not allowed to consume such supplement/food but were asked to maintain their regular habits (eating, physical activity, tobacco, and alcohol consumption). Taking medications or food supplements was not permitted. Furthermore, participants were asked to avoid a heavy meal, alcohol abuse, or strenuous physical exercise during the study. On the day before the study, the participants finished dinner at 9 PM, were hospitalized, and asked to fast overnight (water intake was not restricted). The following morning, after confirmation of compliance, medical examination, physical examination, vital sign measurement, blood test (hematological/blood biochemical test), and blood draw for a baseline blood sample were performed prior to dosing. Subsequently, the participants were administered the test preparations orally with 150 mL mineral water. Their blood specimens were collected 0.5, 1, 2, 4, 8, and 12 hours after the administration of the preparations. After blood sampling at 4 hours and 12 hours, the participants were fed a rice ball containing pickled plums. After dinner, the participants remained in the hospital; water intake was not restricted during their stay. On the second morning, at 24 hours after dosing, the last blood draw, medical examination, physical examination, and vital sign measurement were performed, and the participants were discharged. The second and third doses were administered in the same manner (Table 1). The sampling times were determined with reference to those of the previous report [7] evaluating CR-033P (pre-dosing and at 0.5, 1, 1.5, 2, 3, 4, 6, 8, and 12 hours post dosing). 

### Safety assessment 

Safety and tolerability of the study treatments were assessed by reporting of adverse drug reactions or adverse events, which were performed at medical examinations, physical examinations, vital sign measurements, and blood tests during the study. 

### Sample collection and preparation 

All blood specimens were drawn in 5-mL blood collecting vessels containing heparin, and immediately placed in an ice bath and protected from light. The vessels were centrifuged at 800 × g for 10 minutes at 4 °C to separate plasma. Plasma samples were frozen at –70 °C until analysis. 

### Determination of plasma curcumin concentration 

The plasma curcumin concentration was measured using a slightly modified version of a previously reported procedure [8]. Briefly, 50 μL of plasma sample was transferred to a 10 mL glass tube. Thereafter, 100 μL of 0.1 M sodium acetate buffer (pH 5.0), 10 μL of β-glucuronidase aqueous solution (ca. 68,000 units/mL), and 10 μL of internal standard solution (mepronil 20 ng/mL in 50% methanol) were added. The mixture was allowed to react at 37 °C for 1 hour to hydrolyze the curcumin conjugates. Thereafter, chloroform (0.5 mL) was added. The sample was vortexed for 10 seconds, subjected to 15 minutes of sonication, and then centrifuged at 15,000 × g for 5 minutes at 4 °C to form organic and aqueous layers. The extraction was repeated twice, and the combined organic layers were transferred to a 1-mL tube and evaporated to dryness using a centrifuge concentrator. The dried sample was re-dissolved in 100 μL of 50% methanol solution and centrifuged at 15,000 × g for 5 minutes at 4 °C. 50 µL of the supernatant were injected into the HPLC-MS/MS system (LCMS 8045, Shimadzu, Kyoto, Japan). 

The calibration curve used to quantify the amount of curcumin in the samples was prepared as follows. Briefly, 10 μL of 20 ng/mL of mepronil 50% (v/v) methanol solution and 10 μL of each of curcumin 50% (v/v) methanol solutions (curcumin concentrations: 0, 50, 500, and 5,000 ng/mL) were added to 50 μL of blank plasma. The obtained standard samples, whose curcumin plasma concentrations were 0, 10, 100, and 1,000 ng/mL, were treated in the same manner as the above clinical sample to obtain the calibration curve. The calibration curve was linear within the range of 10 – 1,000 ng/mL, and the lower limit of quantification was 2.27 ng/mL. 

A HPLC-MS/MS system comprising a Shimadzu 8045 HPLC system with (+) electrospray ionization was used to measure the curcumin concentration. The samples were separated using an Atlantis T3 column (2.1 × 150 mm, 3.0 μm, Waters, MA, USA) and a gradient of mobile phase A (0.1% formic acid/H_2_O) and mobile phase B (0.1% formic acid/acetonitrile). The flow rate was 0.2 mL/min, and the column temperature was 40 °C. The mass spectrometer was operated under multiple reaction monitoring using electron-spray thermal ionization. The transitions (precursor to product) were m/z 369.1 → 177.2 (m/z) for curcumin and 270 → 119(m/z) for mepronil. 

### Pharmacokinetic assessment and statistical analysis 

The primary parameters were total plasma curcumin concentration area under the time curve (AUC_0–24h_, AUC_0–12h_, and AUC_0–24h_ dose-adjusted) and maximum plasma concentration (C_max_). Concentrations below the lower limit of quantification (2.27 ng/mL) were recorded as 0. AUCs were calculated using the trapezoidal method, and the C_max_ was the highest measured value. After logarithmic conversion of the measured plasma concentration, analysis of variance (ANOVA) for 3-period crossover design was performed. 

The statistical model of ANOVA includes factors accounting for the following sources of variation: sequence, subjects nested in sequences, period, preparation, and interaction between period and preparation. The factor of subjects nested in sequences is a random effect and used as the between-subject error when carry-over effect (or the factor of sequence) is tested. Other factors are fixed effects. Carry-over effect and the interaction of bioavailability (BA) parameters were assumed absent as they were not statistically significant. 

Further, the least-squares mean value and 90% confidence interval (CI) of the difference between each preparation were obtained. The point estimate of the geometric mean value ratio between each sample and the 90% CI were derived using the inverse log transformation of the calculated least squares mean value and its 90% CI. 

The bioequivalence of the obtained results was evaluated with reference to the “Guideline for bioequivalence studies of generic products (PMDA, Japan: March 19, 2020)”, which served as a reference parameter. The time to reach the maximum plasma concentration (t_max_), which was calculated using the measured value (unconverted value), and plasma concentration half-life (T_1/2_), which was calculated by estimated elimination rate constants, were used and analyzed in the same manner as the primary parameters. All analyses were performed using SAS software version 9.4. 

## Results 

### Participants and safety 

24 participants were enrolled; the cohort included 8 men and 16 women, with mean age of 43.8 (20 – 59) years. The mean body weight was 57.4 (41.9 – 68.8) kg, mean height was 1.69 (1.53 – 1.74) m, and mean BMI was 21.4 (16.6 – 26.0) kg/m^2^. 

The target population for the safety analysis was 24 participants. However, the pharmacokinetic analysis target population was 22 participants; 1 participant dropped out at their request after the start of the first period, and 1 discontinued the study at the doctor’s discretion after the start of the third period (Figure 1). 

No side effects were observed during the study period; however, adverse events included headache, nausea, and vomiting in 2 and 3 cases, respectively. These events were determined by the investigator to have no causal relationship with the test preparation. 

### Pharmacokinetic and statistical analyses 

**Figure 2.**
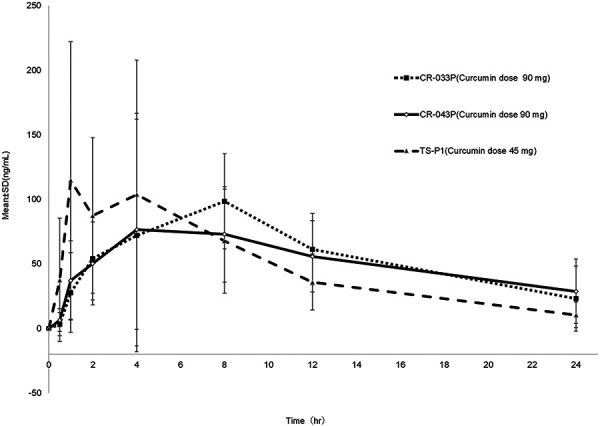
Linear plot of mean plasma concentrations vs. time curves of total curcumin to healthy, adult, human subjects.

The time course of the mean plasma total curcumin concentration over time is shown in Figure 2. 

Table 2 summarizes the AUC_0–24h_, AUC_0–12h_, AUC_0–24h_ dose adjusted, C_max,_ t_max_, and T_1/2_ values of the 3 preparations. TS-P1 and CR-043P showed similar AUC_0–24h_ and AUC_0–12h_ to CR-033P, while the C_max_ of TS-P1 was higher than that of CR-033P and CR-043P. In the comparison of AUC_0–24h_ dose adjusted, the TS-P1 value was significantly larger than either 033P or 043P, and the mean value was almost doubled. The t_max_ results revealed that the absorption rate of TS-P1 was faster than that of CR-033P and CR-043P. Meanwhile, T_1/2_ of TS-P1 was shorter than that of CR-033P and CR-043P. 

Table 3 shows the point estimate of the geometric mean value ratio between each preparation and the 90% CI of C_max_ (as an index of the rate of absorption) and AUCs (as an index of the extent of absorption). 

TS-P1 had a slightly lower AUC_0–24h_, similar AUC_0–12h_, and significantly higher C_max_ than CR-033P. The AUC_0–12h_ ratio was within the bioequivalence (0.80 – 1.25). TS-P1 showed similar AUC_0–24h_, higher AUC_0–12h_, and significantly higher C_max_ than CR-043P. The AUC_0–24h_ ratio met the bioequivalence criteria. CR-043P had slightly lower AUCs and C_max_ values than CR-033P. The dose adjusted area did not meet the 80 – 125% confidence interval. 

A post-hoc power analysis was performed to find out whether the planned sample size had the power to detect bioequivalence for the primary parameters. The powers of AUC_24h_ and AUC_12h_ were 71.0% and 76.6% each. The power of C_max_ was 38.3%. 

## Discussion 

The first objective of the present study was to compare the curcumin PK profiles of a newly developed TS-P1 composition (45-mg dose) with those of the existing CR-033P composition (90-mg dose). Thereafter, the aim was to compare the profile of the CR-043P composition (90-mg dose), an alternative for CR-033P, with the CR-033P composition. The AUC of TS-P1 was similar to that of CR-033P, while its C_max_ was higher than that of CR-033P. In contrast, CR-043P had slightly lower AUCs and C_max_ than CR-033P. 

The pharmacokinetics of CR-033P 90 mg was recently evaluated by Chung et al. [7], who obtained point estimate ratios of C_max_ and AUC_0–12h_ of 20.5-fold and 42.6-fold, respectively, compared to unformulated curcumin. In this study, the point estimate of TS-P1 (45 mg) was 1.54-fold at C_max_ and 1.02-fold at AUC_0–12h_ compared to those of CR-033P 90 mg. TS-P1 is thought to have an approximate 85.2-fold bioavailability relative to that of curcumin powder. The two studies were separate trials performed at different locations, with different analytical methods and participants. Furthermore, the measured plasma concentration values were found to vary. However, when TS-P1 and CR-033P were compared, the oral bioavailability comparison with unformulated curcumin could be further expanded. 

The curcumin in TS-P1 was amorphous, while that in CR-033P and CR-043P was submicron-like crystalline. Amorphous forms are, by definition, non-crystalline materials, and their structure can be considered similar to that of a frozen liquid. Amorphous forms are thermodynamically unstable and serve as the most energetic forms of a material, which may result in higher solubility or a higher dissolution rate. Few reports have been published on the oral absorption of amorphous curcumin. Pawar et al. [9] studied the oral absorption of amorphous curcumin in rats. A dose of 250 mg/kg resulted in a C_max_ of 70 ng/mL (t_max_ = 15 minutes), which was twice that of crystalline curcumin. Although the absorption of the amorphous material was rather rapid, the oral bioavailability (AUC) was improved by only 1.45-fold relative to the control; thus, no significant difference was found [9]. Sunagawa et al. [10] recently studied the bioavailability of amorphous preparations in humans and reported that the new formulation was superior to that of theracurmin (CR-031P 30-mg capsule). However, information on the statistical analysis of the differences between formulations is limited. 

Table 3 shows that the AUCs and C_max_ of CR-043P were slightly lower than those of CR-033P; however, the difference was not significant. Changing a small amount of the dispersant from gum ghatti to modified starch is unlikely to affect the absorption. Accordingly, the above observation seemed reasonable. 

## Conclusion 

A randomized double-blind crossover study (3 groups, 3 periods) was conducted with single oral curcumin-containing preparations, including a newly developed preparation (TS-P1 capsule (45-mg dose)) and two theracurmin products (CR-033P capsule (90-mg dose) and CR-043P capsule (90-mg dose)), which were administered to healthy adult participants. The pharmacokinetics of these preparation were then compared. TS-P1 tended to have a lower AUC_0–24h_ than CR-033P; however, its AUC_0–24h_ displayed bioequivalence to that of CR-043P. The AUC_0–24h_ of CR-043P tended to be slightly lower than that of CR-033P. Furthermore, the AUC_0–12h_ of TS-P1 displayed bioequivalence to that of CR-033P, but was higher than that of CR-043P. CR-043P tended to have a lower AUC_0–12h_ than CR-033P. A statistically significant difference (p < 0.001) in C_max_ was found between the preparations. TS-P1 tended to have a higher C_max_ than CR-033P and CR-043P (~ 1.5-fold and ~ 1.8-fold, respectively), while CR-043P tended to have a slightly lower C_max_ than CR-033P (~ 1.1-fold). Based on the results, TS-P1 (45-mg dose), a newly developed amorphous curcumin preparation, seemed to display similar curcumin oral availability to CR-033P (90-mg dose) and CR-043P (90-mg dose) but higher curcumin plasma concentration than the other preparations. 

## Authors’ contribution 

Research design: YKu and YKa; material preparation: AH, CK, and YM; document preparation: AH and YKa; experiments: AH, CK, and YM; clinical studies: FY; supervision of the clinical studies: KS; statistical analysis and interpretation of the data: YKa; writing of the manuscript: AH, YKa, CK, and YM. 

## Conflict of interest 

AH and YKu are employees of Theravalues Corporation. The other authors have no conflict of interest to declare. 

## Funding 

This study was sponsored by Theravalues Corporation. 

**Table 1.** Overall study design.

	Screening	Allocation	Before dosing	Dosing (1^st^, 2^nd^, and 3^rd^ term)							
				0.0 h	0.5 h	1 h	2 h	4 h	8 h	12 h	24 h
Informed consent	●										
Background survey	●										
Medical examination	●		●							●	●
Physical examination	●		●								
Vital sign measurement	●		●					●		●	●
Blood test	●		●								●
Blood draw			●		●	●	●	●	●	●	●
Urine test	●										
Behavior recording				<----	-----	-----	-----	-----	-----	-----	---->
Meal								●		●	

**Table 2.** Pharmacokinetic parameters after single oral dose of curcumin preparations.

Preparation	TS-P1 capsule						CR-043P capsule						CR-033P capsule					
	AUC_0–24h_	AUC_0–12h_	AUC_0–24h_ dose adjusted	C_max_	t_max_	T_1/2_ (complete case)	AUC_0–24h_	AUC_0–12h_	AUC_0–24h_ dose adjusted	C_max_	t_max_	T_1/2_ (complete case)	AUC_0–24h_	AUC_0–12h_	AUC_0–24h_ dose adjusted	C_max_	t_max_	T_1/2_ (complete case)
	(ng/mL×h)	(ng/mL×h)	(ng/mL×h)	(ng/mL)	(h)	(h)	(ng/mL×h)	(ng/mL×h)	(ng/mL×h)	(ng/mL)	(h)	(h)	(ng/mL×h)	(ng/mL×h)	(ng/mL×h)	(ng/mL)	(h)	(h)
Mean	1,168.88	889.76	25.98	179.91	2.18	6.23	1,247.32	739.46	13.86	104.90	6.59	10.18	1,345.39	837.56	14.95	108.60	6.50	8.89
SD	635.95	491.80	14.13	109.67	1.33	2.70	615.40	406.49	6.84	85.79	5.22	3.86	580.35	397.43	6.45	54.68	2.65	4.07
Geometric mean	1,036.81	791.16	23.04	152.63	1.82	5.72	1,092.63	648.13	12.14	85.95	4.88	10.03	1,235.61	762.92	13.73	97.59	5.76	8.60
Median	953.62	724.56	21.19	162.78	2.00	5.85	1,179.90	675.37	13.11	96.24	4.00	10.46	1,307.31	795.71	14.53	100.83	8.00	8.16
Min.	511.97	414.89	11.38	55.73	1.0	2.38	204.41	156.35	2.27	18.03	1.0	5.42	503.12	397.94	5.59	43.69	1.0	4.12
Max.	2,756.17	2,202.85	61.25	500.37	4.0	14.37	2,894.74	2,111.02	32.16	454.30	24.0	18.25	3,031.31	2,116.61	33.68	281.91	12.0	15.97

**Table 3.** 90% confidence intervals of ratio of geometric means for AUCs and Cmax.

Comparison	Geometric least squares mean ratio			
	(90% confidence interval)			
	AUC_0–24h_	AUC_0–12h_	AUC_0–24h_ dose adjusted	C_max_
TS-P1 to CR-033P	0.83 (0.70 – 0.99)	1.02 (0.87 – 1.21)	1.67 (1.40 – 1.98)	1.54 (1.23 – 1.93)
CR-043P to CR-033P	0.88 (0.74 – 1.05)	0.84 (0.72 – 0.99)	0.89 (0.74 – 1.05)	0.88 (0.70 – 1.10)
TS-P1 to CR-043P	0.94 (0.80 – 1.12)	1.21 (1.03 – 1.43)	1.89 (1.59 – 2.24)	1.75 (1.40 – 2.20)

## Supplemental material

Supplemental material Supplemental Tables 1 and 2.
