# Supplementary material for: Comparative pharmacokinetics of new curcumin preparations and evidence for increased bioavailability in healthy adult participants
Source: Int J Clin Pharmacol Ther. 2022 Oct 24;60(12):530–8. doi: 10.5414/CP204257 (PMC9685553; doi:10.5414/CP204257)
Supplement: Supplemental material [file intjclinpharmacol-60-530-S01.pdf]

Supplemental Table 1. Inclusion criteria

1)Who are aged 20–59 years of either sex Japanese
2)Who are capable of visiting the hospital according to the schedule and of being hospitalized for three days at each term of the study
3)Who provide written informed consent prior to the study

Supplemental Table 2. Exclusion criteria

1)Who have been regularly consuming any curcumin–containing food

2)Who have been regularly consuming any specific health food, functional food or health food

3)Whose body weight are no more than 40kg or no less than 100kg, or whose BMI are no less than 31

4)Who are with current or history of severe heart disease, pulmonary disease, digestive disease (esophagus, stomach, small intestine, large intestine, liver, kidney and so on), hypertension, diabetes or malignant tumor,

5)Who are regularly treated with prescription drugs,

6)Who are pregnant or lactating at screening, or planning to be pregnant during the study

7)Who have or may have allergy to the investigational preparations, or may have severe allergy to other foods and drugs

8)Who had a history of more than 200mL whole blood drawing within 4 weeks prior to the screening, or of more than 400mL whole blood sampling or more than 600mL ingredient blood sampling within 16 weeks prior to the screening

9)Who have a history of complaining feeling at the blood drawing,

10)Who participate or will participate in another clinical study during this study,

11)Who are judged to be excluded by the responsible doctor
